# A comprehensive comparative genomic analysis revealed that plant growth promoting traits are ubiquitous in strains of *Stenotrophomonas*

**DOI:** 10.3389/fmicb.2024.1395477

**Published:** 2024-05-16

**Authors:** Yang Zhao, Wen-Jing Ding, Lian Xu, Ji-Quan Sun

**Affiliations:** ^1^Lab for Microbial Resources, School of Ecology and Environment, Inner Mongolia University, Hohhot, China; ^2^Jiangsu Key Lab for Organic Solid Waste Utilization, Educational Ministry Engineering Center of Resource-saving Fertilizers, Jiangsu Collaborative Innovation Center for Solid Organic Waste Resource Utilization, Nanjing Agricultural University, Nanjing, China

**Keywords:** *Stenotrophomonas*, comparative genomic analysis, environmental adaptability, plant growth promotion, horizontal gene transfer

## Abstract

*Stenotrophomonas* strains, which are often described as plant growth promoting (PGP) bacteria, are ubiquitous in many environments. A total of 213 genomes of strains of *Stenotrophomonas* were analyzed using comparative genomics to better understand the ecological roles of these bacteria in the environment. The pan-genome of the 213 strains of *Stenotrophomonas* consists of 27,186 gene families, including 710 core gene families, 11,039 unique genes and 15,437 accessory genes. Nearly all strains of *Stenotrophomonas* harbor the genes for GH3-family cellulose degradation and GH2- and GH31-family hemicellulose hydrolase, as well as intact glycolysis and tricarboxylic acid cycle pathways. These abilities suggest that the strains of this genus can easily obtain carbon and energy from the environment. The *Stenotrophomonas* strains can respond to oxidative stress by synthesizing catalase, superoxide dismutase, methionine sulfoxide reductase, and disulfide isomerase, as well as managing their osmotic balance by accumulating potassium and synthesizing compatible solutes, such as betaine, trehalose, glutamate, and proline. Each *Stenotrophomonas* strain also contains many genes for resistance to antibiotics and heavy metals. These genes that mediate stress tolerance increase the ability of *Stenotrophomonas* strains to survive in extreme environments. In addition, many functional genes related to attachment and plant colonization, growth promotion and biocontrol were identified. In detail, the genes associated with flagellar assembly, motility, chemotaxis and biofilm formation enable the strains of *Stenotrophomonas* to effectively colonize host plants. The presence of genes for phosphate-solubilization and siderophore production and the polyamine, indole-3-acetic acid, and cytokinin biosynthetic pathways confer the ability to promote plant growth. These strains can produce antimicrobial compounds, chitinases, lipases and proteases. Each *Stenotrophomonas* genome contained 1–9 prophages and 17–60 genomic islands, and the genes related to antibiotic and heavy metal resistance and the biosynthesis of polyamines, indole-3-acetic acid, and cytokinin may be acquired by horizontal gene transfer. This study demonstrates that strains of *Stenotrophomonas* are highly adaptable for different environments and have strong potential for use as plant growth-promoting bacteria.

## Introduction

*Stenotrophomonas* is a genus with versatile metabolic capabilities, which belongs to the family Lysobacteraceae in the order Lysobacterales and is a common genus in various environments (Ryan et al., 2009). Many strains of *Stenotrophomonas* have been described as plant growth-promoting (PGP) bacteria owing to their various mechanisms (Singh and Jha, 2017; An and Berg, 2018; Alexander et al., 2019). For example, *S. maltophilia* SBP-9, a strain that simultaneously solubilizes inorganic phosphate and produces indole-3-acetic acid (IAA), gibberellic acid, ACC deaminase, and siderophores, significantly increased the biomass and chlorophyll content of wheat (*Triticum aestivum*) (Singh and Jha, 2017). *Stenotrophomonas* sp. CV83, which produces IAA, ACC deaminase and siderophores, can significantly enhance the growth of chickpea (*Cicer arietinum*) under drought stress conditions (Sharma et al., 2023). *S. maltophilia* SR1, a bacterium that can degrade aromatic hydrocarbons, exhibited PGP properties (Bashandy et al., 2020). Many strains of *Stenotrophomonas* have also been demonstrated to protect plants from phytopathogens via multiple ways. For example, *S. maltophilia* UN1512 can inhibit *Colletotrichum nymphaeae*, a fungal fruit and leaf pathogen, by secreting volatile and non-volatile organic compounds (Alijani et al., 2020). *S. maltophilia* C3 can decrease the incidence of Bipolaris leaf spot caused by the fungal pathogen *Bipolaris sorokiniana* by producing chitinases (Zhang et al., 2001). *S. maltophilia* MB9 can protect plants from multiple pathogens, including *Curvularia* sp., *Aspergillus niger*, *Fusarium oxysporum*, *Diploidia* sp., and *Rhizoctonia solani*, by producing the broad-spectrum antifungal compound dodecanoic acid (John and Thangavel, 2017).

Currently, a substantial amount of comparative genomic research on the species of *Stenotrophomonas* provides new insight on the formation of biofilm (Flores-Treviño et al., 2019), quorum sensing (QS) signaling and quenching (Huedo et al., 2018), genome evolution (Xu et al., 2023), PGP and antibiotic resistance mechanisms (Huang et al., 2018; Ulrich et al., 2021), and bioremediation (Mukherjee and Roy, 2016). For example, a comparison of the genome of the plant-associated strain *S. rhizophila* DSM 14405^T^ with *S. maltophilia* K29a and R551-3 revealed that *S. rhizophila* DSM 14405^T^ possesses unique genes for the biosynthesis and transportation of plant-protective spermidine, plant cell wall-degrading enzymes, and high salt tolerance, but it lacks several critical virulence factors and heat shock genes (Alavi et al., 2014). A comparative genomic analysis of 30 *S. maltophilia* and seven *S. rhizophila* strains revealed that 96 genes, including chitin-binding proteins and mechanosensitive channels protein genes, are unique to *S. maltophilia*, and 59 genes are unique to *S. rhizophila*. The strains within both species have a high potential for biocontrol owing to their production of proteases, chitinases and keratinases, as well as similar PGP properties provided by the biosynthesis of siderophores and spermidine (Pinski et al., 2020). A comparative genomic analysis based on the 14 genomes of *S. maltophilia* revealed that the antibiotic resistance genes were not significantly different between the clinical and environmental strains (Youenou et al., 2015).

Although a substantial amount of comparative genomic research related to *Stenotrophomonas* strains has been conducted, a systematic and comprehensive comparative genome of this genus, particularly comparative information on the PGP properties is still missing. To better understand the PGP mechanisms of the *Stenotrophomonas* strains and their ecological roles in the rhizosphere, a comprehensive comparative genomic analysis based on the genomes of 213 strains of *Stenotrophomonas* was conducted in this study.

## Materials and methods

### Genome sources and analysis of the *Stenotrophomonas* strains

By September 2023, there were 1,206 genome sequences of *Stenotrophomonas* in GenBank. A total of 727 unique genomes were downloaded. The downloaded genome sequences were then evaluated using CheckM (Parks et al., 2015). Only 160 genomes of *S. maltophilia* (>99.5% completeness and < 0.5% contamination) and 53 genomes of other species of *Stenotrophomonas* (>90.0% completeness and contamination <6.0%) were selected for subsequent comparative genomic analyses. More details about the 213 *Stenotrophomonas* genomes are listed in Supplementary Table S1. The global distribution and habitat preference of the genus *Stenotrophomonas* was evaluated using the analytical pipeline Microbe Atlas Project (MAP)^1^ (Matias Rodrigues et al., 2017).

### Pan-genome analysis of *Stenotrophomonas*

All the downloaded genome sequences were annotated using Prodigal (Hyatt et al., 2010). The faa files that were produced were used for pan- and core-genome analyses using the Bacterial Pan Genome Analysis tool (BPGA) pipeline v. 1.3 (Chaudhari et al., 2016). In a pan-genome analysis, the number of accumulated genes that are related to the number of genomes can be predicted by Heaps’ law as $n=a\;.\;x^{b}$ (Tettelin et al., 2008). In the equation, x is the number of genomes, while a and b are fitting parameters. When 0 < b < 1 indicates that the pan-genome is open, and only b < 0 indicates that the pan-genome is closed.

### Phylogenetic analysis and measurements of genomic similarity

The core genome of *Stenotrophomonas* was constructed by aligning 213 genomes using USEARCH with a cut-off value 50% sequence identity. After the resulting core genes were concatenated and aligned using MUSCLE, a phylogenetic tree was constructed based on the concatenated core genes using the neighbor-joining (NJ) method (Chaudhari et al., 2016).

The amino acid sequences of the polyamines, IAA and cytokinin synthases were aligned using ClustalW (Hall, 2013). A phylogenetic tree based on these single coding sequences (CDS) was constructed using MEGA software v. 6.0 (Tamura et al., 2013) with the NJ algorithm (Saitou and Nei, 1987). The tree topology was evaluated using the bootstrap analysis based on 1,000 resampling replicates. The iTOL online server^2^ was used to display the phylogenetic tree (Letunic and Bork, 2021). The ANI values between these strains were calculated using fastANI v. 1.33 (Jain et al., 2018). The dDDH values were calculated using the Genome to Genome Distance Calculator (GGDC 2.5)^3^ (Auch et al., 2010).

### Analysis of COG and metabolism

A COG analysis of the sequences of proteins from the core, accessory and unique gene families was used to categorize them according to the COG database using BPGA (v. 1.3). To ensure the accuracy and completeness of the results, only 33 complete *Stenotrophomonas* genomes were selected for analysis of their primary metabolic profile and the prediction of secondary metabolite biosynthetic genes using KAAS (KEGG Automatic Annotation Server) and the antiSMASH version 6.0 online^4^ (Blin et al., 2021), respectively. The carbohydrate active enzymes (CAZymes) were identified using the HMMER method of the dbCAN online server^5^ (Zheng et al., 2023) with the e-value thresholds ≤1e^−15^ and coverage >0.35.

### Search for functional genes in the genomes

The genes related to PGP, oxidative and osmotic stresses, as well as resistance to heavy metals, were firstly searched in 33 complete genomes, and the sequences obtained were compared with the reference sequences in GenBank to determine the correctness of the sequences. Subsequently, the sequences that were obtained were used as the reference sequences to search their counterparts in the remaining genomes using BLAST.^6^ Only sequences that had >40% identity with the reference sequences indicated that the genome had the gene (Zhao et al., 2023).

The antibiotic resistance genes were identified using resistance gene identifier (RGI) of the CARD^7^ (Alcock et al., 2019), and the sequences with Perfect, Strict and Loose hits identities >70% were selected for further analysis.

### Identification of mobile elements in the genome

Integrated prophages were identified using the PHASTER online server^8^ (Arndt et al., 2016). The predicted prophages with completeness score > 90 were thought to be intact; those with a completeness score of 60–90 were questionable prophages, and those with a completeness score < 60 were considered to be incomplete prophages. The genomic islands (GIs) of the genomes were predicted using the IslandViewer 4 online server^9^ with the IslandPick (Langille et al., 2008), SIGI-HMM (Waack et al., 2006), IslandPath-DIMOB (Hsiao et al., 2003), and Islander (Hudson et al., 2014) methods (Bertelli et al., 2017).

To learn the original source of the biosynthetic genes for polyamines, IAA, and cytokinin, the topologic differences of the phylogenetic tree between the single genes and the core genome were compared. The DNA genomic G + C content of the single genes and the whole genomes were compared (Syvanen, 1994; Garcia-Vallve, 2000).

## Results

### General features and phylogenetic analysis of the *Stenotrophomonas* strains

Among the 213 strains, 78 were isolated from environmental habitats, such as soil, plants, sludge, water, and biofilm reactors, and 135 were from materials from clinical settings, such as urine, blood, sputum, wounds, lungs, and animals among others. The species *S. maltophilia*, *S. rhizophila*, *S. indicatrix*, *S. pavanii* and *S. geniculata* can be found in both clinical and environmental habitats, while *S. acidaminiphila*, *S. lactitubi*, *S. nitritireducens*, and *S. bentonitica* were only found in environmental habitats.

The genome size of the 213 strains of *Stenotrophomonas* ranged from 3.50 to 5.12 Mb with a G + C content of 63.8–69.3%. The number of CDS ranged from 3,099 to 5,180. The strains with the largest genome and CDS numbers were *S. geniculata* NWUBe21, which was isolated from seeds, and *S. geniculate* E119, which was isolated from feces, while the smallest was *S. pictorum* JCM 9942^T^, which isolated from soil. *S. acidaminiphila* Au-Ste40, which was isolated from soil, contained the highest content of genomic DNA G + C, while *S. nitritireducens* 2001 that was isolated from lake sediments had the lowest.

**Figure 1:**
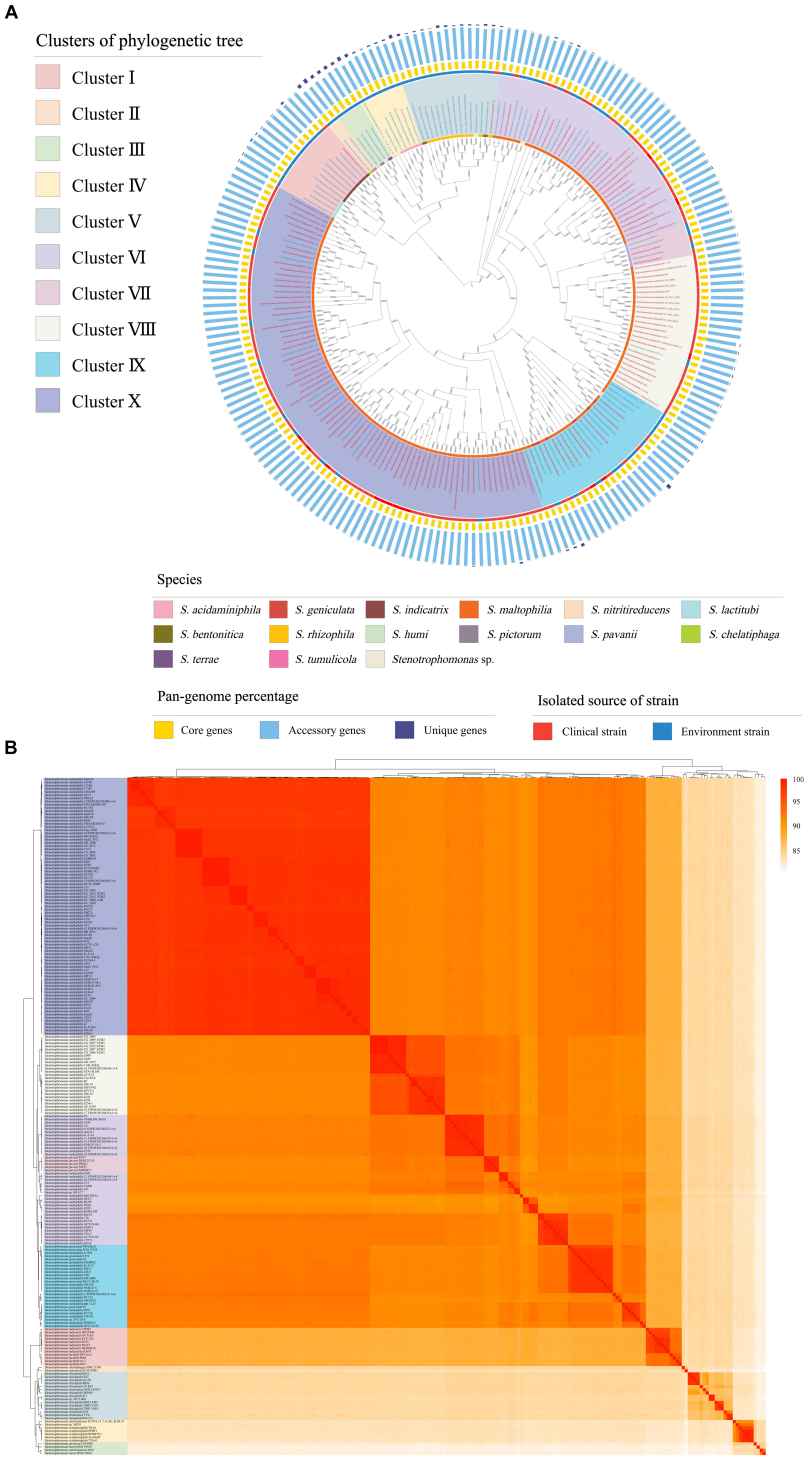
**(A)** Phylogenetic tree constructed based on core genomes. Clinical strains are marked with red font and environmental strains with blue font. The three colors in the bar graph represent the percentage of core, accessory, and unique genes in the pan-genome. **(B)** Pairwise average nucleotide identity comparison between the genomes of 213 strains of *Stenotrophomonas*. The ANI values were used to construct a dendrogram and a heatmap with the average linkage method and Euclidean distance used for clustering and correlation analyses, respectively.

The phylogenetic tree based on core genes showed that the 213 strains of *Stenotrophomonas* can be subdivided into 10 clusters (Figure 1A). Among them, Clusters I, II, III, IV, V and IX are composed of two (*S. indicatrix* and *S. lactitubi*), two (*S. chelatiphaga* and *S. tumulicola*), four (*S. terrae*, *S. nitritireducens*, *S. pictorum* and *S. humi*), two (*S. acidaminiphila* and *S. nitritireducens*), two (*S. bentonitica* and *S. rhizophila*) and two (*S. maltophilia* and *S. geniculate*) species, respectively. Clusters VI, VIII, and X all consist of *S. maltophilia*, while Cluster VII only comprises strains of *S. pavanii*. The ANI values between all the strains tested were higher than 81%, which confirmed that the 213 strains of *Stenotrophomonas* belonged to a single genus (Supplementary Table S2). The topology of the tree based on ANI values was similar to that based on the core genome with only minor differences (Figure 1B). Cluster VII formed a clade with Cluster VI in the ANI phylogenetic tree.

There were 14 different *Stenotrophomonas* species found in 10 clusters. Among them, *S. lactitubi*, *S. indicatrix*, *S. acidaminiphila*, *S. nitritireducens*, *S. bentonitica*, *S. rhizophila*, *S. maltophilia*, *S. pavanii*, and *S. geniculata* contain multiple strains. The ANI values of strains within the same species of *S. lactitubi* (95.1–96.9%), *S. indicatrix* (96.0–98.8%), *S. acidaminiphila* (97.1–98.9%), and *S. pavanii* (98.6–98.7%) were > 95.0% (Supplementary Table S2), which indicated that the non-type strains of these species were accurately classified. In contrast, the values between some non-type strains within the other species of *Stenotrophomonas* compared to their type strain were lower than the threshold value for the delineation of a species (ANI value <95% and dDDH value <70%), which indicated that these strains had been misidentified. For example, strains of *S. nitritireducens* are present in both Clusters III and IV, and some *S. maltophilia* strains tightly clustered with *S. geniculata* in Cluster IX. Strains 2001 and SCN18_13_7_16_R1_B_68_91, which are currently both identified as *S. nitritireducens*, are scattered in Clusters III and IV, respectively. The ANI and dDDH values of strain 2001 to the type strain of *S. nitritireducens* were only 84.9 and 27.0%, respectively, indicating that it had been misidentified. Nine of 12 *S. rhizophila* strains had <90% ANI and < 35% dDDH values to the type strain of *S. rhizophila* (DSM 14405^T^), which suggested that these nine strains should not be identified as *S. rhizophila*. Strains of *S. maltophilia* were widely scattered in Clusters VI, VIII, IX, and X. Only those in Cluster X shared high ANI (>95%) and dDDH (>70%) values with the type strain, while the two values of the other three cluster strains with *S. maltophilia* NBRC 14161^T^ ranged from 91.2–94.0% and 41.0–52.0%, respectively. The low values indicate that these strains were misidentified as *S. maltophilia* (Supplementary Table S2). Among them, the strains in Cluster VIII share high values of ANI and dDDH with each other and low values with the other strains, indicating that they belong to an unidentified novel species, while the ANI and the dDDH values between the strains in Cluster VI were 90.7–99.9% and 39.5–99.5%, respectively, indicating that the strains in this cluster can be further divided into two or more different species, and the classification of the Cluster IX strains was similar to that of Cluster VI.

### Analyses of the pan-genome and COG distribution

**Figure 2:**
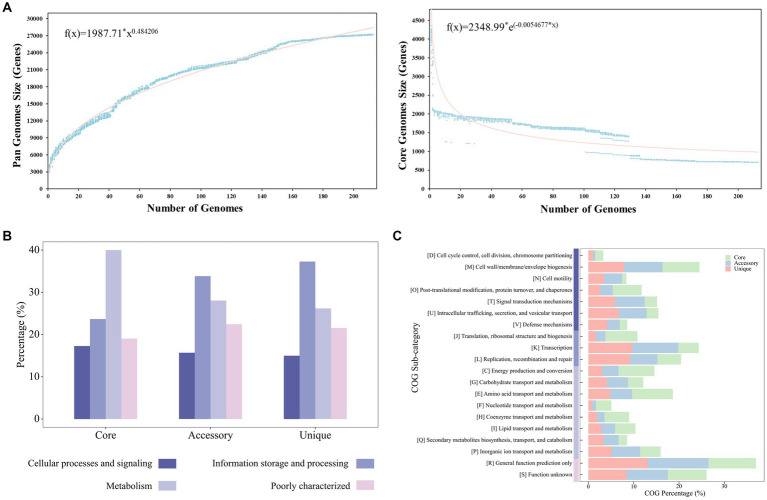
**(A)** Pan-genome and core genome plots of the 213 strains of *Stenotrophomonas*. The plot shows the equations fitting the total and core gene families, as well as how the number of gene families increase and decline in the pan and core genome with each consecutive addition of a *Stenotrophomonas* genome. The functional proportions of core, accessory and unique genes in COG categories **(B)** and COG sub-categories **(C)**.

The pan-genome of the 213 *Stenotrophomonas* strains contained 27,186 gene families. There were 710, 15,437, and 11,039 core, accessory, and unique genes, respectively. Each of the 213 *Stenotrophomonas* genomes were composed of 15.1–24.3% core genes and 69.0–83.5% accessory genes, while the unique genes only accounted for 0–11.1% of the genomes. According to Heaps’ law, the pan-genome of *Stenotrophomonas* was open and increasing (*b* = 0.48) (Figure 2A).

The most common categories of COGs in the core genome of *Stenotrophomonas* (40.0%) were related to metabolism, while those of the accessory genomes (33.8%) and unique genomes (37.3%) were both related to information storage and processing (Figure 2B). The most abundant components of the core genome were related to the maintenance of primary cellular processes, such as class E (9.0%; amino acid transport and metabolism), class C (7.9%; energy production and conversion) and class J (7.1%; translation, ribosomal structure and biogenesis) (Figure 2C). The percentages of classes E, C, and J in the accessory and unique genomes were 4.9 and 4.8%, 3.6 and 3.1%, and 2.1 and 1.6%, respectively. In contrast to the core genome, the accessory and unique genomes were primarily related to the functions of adapting to environmental niches. They were the most enriched in class K (transcription), class U (intracellular trafficking, secretion, and vesicular transport), class T (signal transduction mechanisms), class Q (secondary metabolites biosynthesis, transport, and catabolism) and class V (defense mechanisms), which accounted for 10.2 and 9.7% (4.5%), 6.1 and 6.7% (2.6%), 6.6 and 5.9% (2.7%), 3.2 and 3.4% (1.9%), as well as 2.8 and 4.2% (1.6%), respectively (Figure 2C).

### Main metabolism and secondary metabolites in the strains of *Stenotrophomonas*

**Figure 3:**
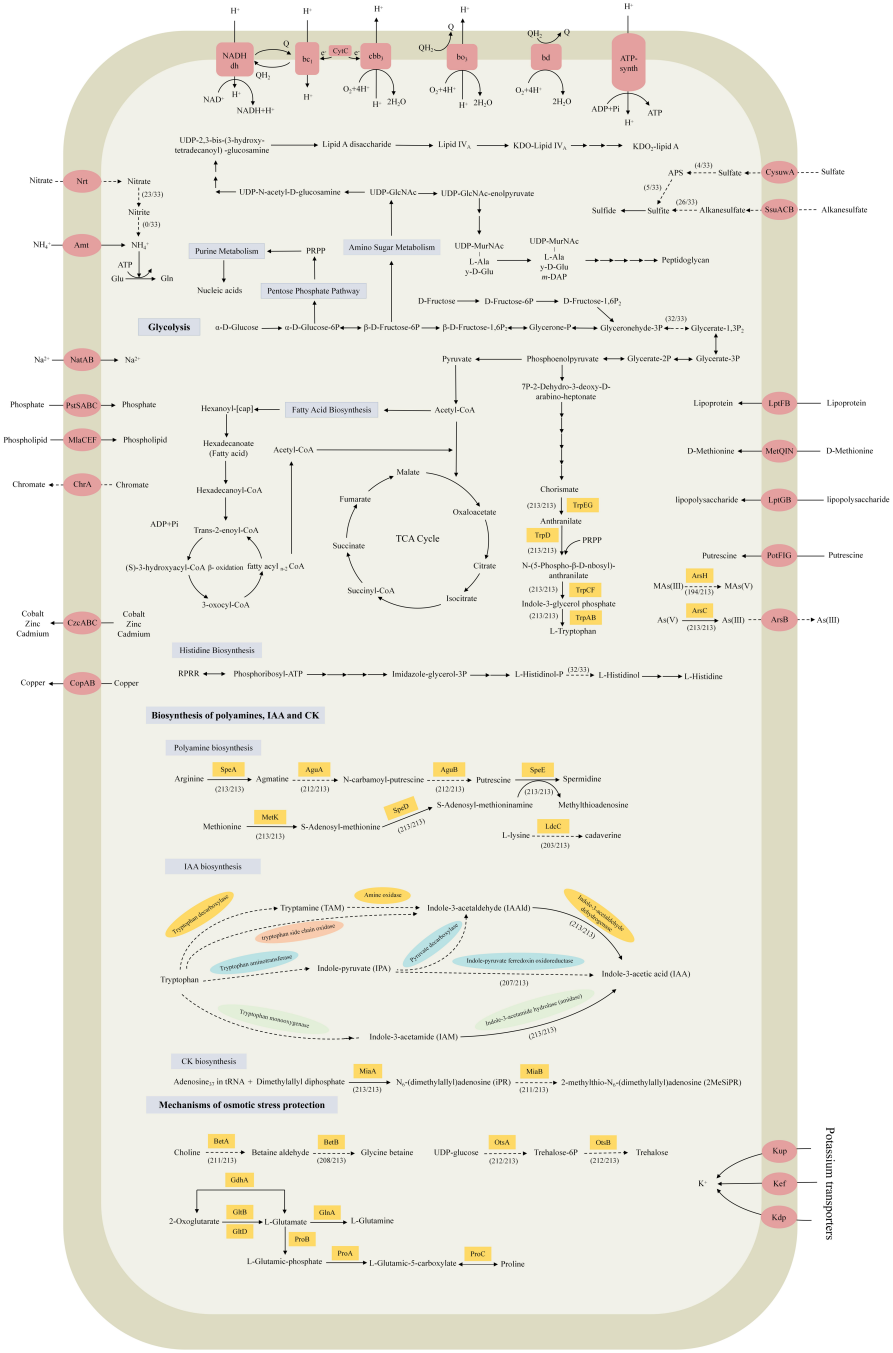
Prediction of the central metabolic potential of 33 complete genomes of *Stenotrophomonas* strains, as well as the possible biosynthetic pathway for phytohormones and the mechanism of hyperosmolar adaptation of the 213 strains. Solid lines, pathways common to all bacteria; dashed lines, partial presence.

All 33 strains with complete genome sequences harbored the intact pentose phosphate pathway, fructose metabolism pathway, tricarboxylic acid cycle (TCA cycle), fatty acid, lipopolysaccharide, and peptidoglycan biosynthetic pathways. Only *S. geniculata* E119 lacked an intact glycolysis pathway owing to a deficiency in the glyceraldehyde 3-phosphate dehydrogenase (*gapA*) gene that converts glyceraldehyde-3P to glycerate-1,3P_2_ (Figure 3; Supplementary Table S3). In terms of nitrogen metabolism, all 33 strains harbored the Amt family ammonia transporter gene, which is responsible for the uptake of ammonia. In particular, *S. indicatrix* MGMM10, *S. nitritireducens* 2001, *S. maltophilia* strains PSKL2, FDAARGOS_92, and FDAARGOS_507, and *S. rhizophila* strains DSM 14405^T^, Bl2-2, JC1, PI-27, and QL-P4 lacked both nitrate reductase and nitrite reductase, while the other 23 strains only lacked nitrite reductase. This indicated that all these strains cannot utilize nitrate as a nitrogen source. The metabolism of sulfur also varies between strains, and only *S. acidaminiphila* strains T0-18, SPDF1, and T25-65, and *S. nitritireducens* 2001 had an intact sulfate reduction pathway, while the remaining 29 strains lacked the genes involved in the conversion of sulfate to sulfite. However, they harbored the genes to convert sulfite to sulfide. In terms of the biosynthesis of amino acids, all 33 strains of *Stenotrophomonas* can synthesize 20 types of amino acids. There was only one exception. *S. geniculata* E119 cannot synthesize histidine because it lacks histidinol-phosphatase. In addition, the phosphate-specific ABC transporter complex PstSABC and the two-component system PhoRB were identified in the core genome. This indicates that the strains of *Stenotrophomonas* can take up inorganic phosphate and regulate phosphate homeostasis.

Bacterial chemotaxis is an important prelude to metabolism, competition, symbiosis, infection and other ecological interactions in bacterial communities (Kato et al., 2008). The genomic analysis revealed that all 33 strains of *Stenotrophomonas* had the genes for flagellar proteins and the chemotaxis signaling system. In particular, all 33 strains of *Stenotrophomonas* harbored the *flg* operon (flagellar motif), the *fli* cluster, and *flhAB* that encodes the structural proteins for the flagella, as well as the *motAB* and *filA* genes that encode the quorum unit MotAB and the sigma factor FliA, respectively. The chemotaxis genes had the *cheABYWR* and *mcp* genes that encode the chemotaxis and methyl-accepting chemotaxis proteins, respectively. The two-component system DesK-DesR genes, which regulate the biosynthesis of fatty acid desaturase (Des), was found in all 33 strains (Beranová et al., 2010). In addition, the genes for DNA mismatch repair, glutathione and folate biosynthesis, peroxidase, and resistance to cationic antimicrobial peptides (CAMP) and β-lactams were also found in the core genome, which enables the strains of *Stenotrophomonas* to adapt to complex and variable environments more efficiently.

Secondary metabolites can enhance the environmental adaptability of the strains and provide them with an evolutionary advantage (O’Connor, 2015). A total of 149 smBGCs of 14 major types were predicted in the 33 *Stenotrophomonas* genomes, and each strain contained three to seven smBGCs (Supplementary Table S4). Among them, *S. maltophilia* strains JZL8 and ISMMS2 had the highest number of smBGCs with seven, while each strain of *S. nitritireducens* 2001, and *S. rhizophila* strains Bl2-2, JC1, and QL-P4 had only three smBGCs. In detail, 55 RiPP-like smBGCs were found in 32 strains of *Stenotrophomonas* (Supplementary Figure S1), and 22 of the clusters were similar to the entolysin BGCs, which encode a cyclic lipopeptide (CLP) that can be fatal to bacteria, fungi, and viruses by disrupting their membranes (Oni et al., 2019). The clusters responsible for the biosynthesis of aryl polyenes (APEs), which help bacteria to evade the host immune system (Lee et al., 2021a,b), were found in 31 strains except for *S. maltophilia* CYZ and *S. rhizophila* JC1. The NRP-metallophore/NRPS heterozygous clusters were found in 29 *Stenotrophomonas* strains, and all of them were similar to the 2,3-dihydroxybenzoylserine (DHBS) BGCs, which is a cluster that is responsible for the biosynthesis of catecholate siderophore enterobactin (Ahire et al., 2011). In addition, some smBGCs are strain-specific. For example, the cluster responsible for ranthipeptide, lanthipeptide-class-i and lanthipeptide-class-iii biosynthesis was only detected in *S. maltophilia* strains CSM2, ISMMS2, and JV3, respectively.

### Diversity of CAZymes in *Stenotrophomonas* strains

Carbohydrates are the primary source of carbon for most microbes. Carbohydrate active enzymes (CAZymes), a class of enzymes that is involved in the metabolism of carbohydrates, is not only involved in the biosynthesis and degradation of biopolymers but also in the formation of bacterial biofilms and the glycosylation of proteins and lipids (Benini, 2020). A total of 118 different CAZyme genes family were identified in the 213 *Stenotrophomonas* genomes, including 63 glycoside hydrolases (GHs), 21 glycosyltransferases (GTs), 11 carbohydrate esterases (CEs), 9 polysaccharide lyases (PLs), 8 auxiliary activities (AAs), and 6 carbohydrate-binding modules (CBMs) (Supplementary Figure S2). *S. rhizophila* CFBP 13503 was identified as harboring the most CAZyme genes at 124, while *S. pictorum* JCM 9942^T^ had the fewest (68). The CAZyme number of the strains from clinical sites (69 to118, average 106) was similar to that of the strains from the environmental strains (68 to 124, average 104).

In particular, this study explored the CAZymes involved in the degradation of complex polysaccharides. Lignocellulolytic enzymes can be subdivided into cellulases, hemicelluloses, and lignin-modifying enzymes. Most cellulases and hemicelluloses are members of the GHs family, while the lignin-modifying enzymes are generally in the AAs protein family (López-Mondéjar et al., 2016; Zhou et al., 2018). Among them, all 213 strains produced GH3-family enzymes that degraded cellulose, while only some of the 213 strains harbored GH5- (45 strains), GH8- (44 strains), GH1- (19 strains), GH9- (4 strains) and GH74-family (5 strains) enzymes involved in the degradation of cellulose. The hemicellulose hydrolases produced by these species primarily include GH2 (211 strains), GH31 (209 strains), GH10 (162 strains), GH43 (100 strains), GH16 (13 strains) and GH39 (2 strains) families. Lignin-modifying enzymes were primarily represented by the families for laccase-like multi-copper (AA1) and lignin-modifying peroxidases (AA2) (Ma et al., 2021). Genes for the AA1 family were found in all 213 genomes, while AA2 was not found in any of them. In addition, the GH18, GH19, and GH20 families, which are responsible for the degradation of chitin (Swiontek Brzezinska et al., 2013), were detected in 186, 61, and 210 strains, respectively. The analysis revealed that the strains of *Stenotrophomonas* produced a large number of CAZymes involved in the degradation of polysaccharides, which indicates that these strains can easily obtain carbon and energy from their environment.

### The ability to adapt to abiotic stresses

**Figure 4:**
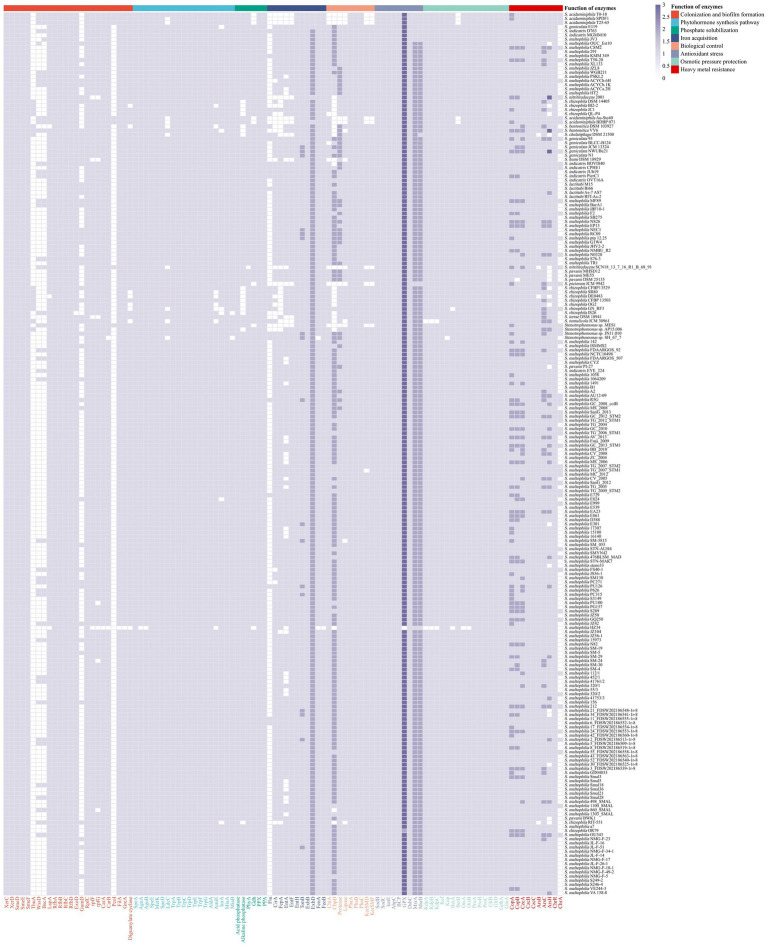
Distribution of enzymes with functions related to plant growth promotion and environmental adaptation in 213 strains of *Stenotrophomonas*. Enzymes associated with colonization and biofilm formation, XerC, XerD, SmeDEF, ThuA, WssD, BscA, LapA, RfbA, RfbB, RfbC, RfbD, ExoD, GumD, RpfC, RpfF, RpfG, CarA, CarB, PcoI, GreA, FilA, and diguanylate cyclase; polyamine synthases, SpeA, AguA, AguB, SpeE, MetK, SpeD, LdcC; IAA synthases, TrpAB, TrpC, TrpD, TrpEG, TrpF, IorA, AmiE, and AldA; CK synthases, MiaA and MiaB; phosphate-solubilizing enzymes, acid phosphatase, alkaline phosphatase, PhyA, GDH, exopolyphosphatase, and inorganic pyrophosphatase; iron acquisition proteins, Fiu, CirA, FepA, EntA, EntF, EntH, TonB, ExbB, ExbD, FeoA, and FeoB; biological control enzymes, ChiA, CbpD, protease, lipase, PhaA, PhaB, PhaC, KerSMD, and KerSMF; antioxidant stress kinases, SodB, SodC, KatE, AhpC, BpC, GPX, DsbC, MrsA, and MrsB; potassium ion transporters, KdpA, KdpB, KdpC, Kef, Kup; compatible solute synthases, BetA, BetB, OtsA, OtsB, ProA, ProB, ProC, GltB, GltD, GdhA, and GlnA; heavy metal resistance enzymes, CopA, CopB, CzcA, CzcB, CzcC, ArsB, ArsC, ArsH, ChrA, and ChrR.

To survive in environments, particularly those that are extreme, microbes have evolved the ability to surmount oxidative stress and osmotic pressure (Zhao et al., 2023). Among these, oxidative stress is the result of an imbalance between the production of reactive oxygen species (ROS) and the ability of biological systems to detoxify them (Chautrand et al., 2022). Bacteria use two primary mechanisms to detoxify the ROS. One is to scavenge the ROS using superoxide dismutase (SOD) and catalase (CAT) (Arts et al., 2015). The *sodB* and *sodC* genes, which are responsible for catalyzing the decomposition of superoxide anion (O_2_^−^) to O_2_ and hydrogen peroxide (H_2_O_2_), were found in all 213 strains of *Stenotrophomonas* (Figure 4). The *katE*, *ahpC* and *bcp* genes, which are responsible for the conversion of H_2_O_2_, were also found in all the strains. The *gpx* gene encodes glutathione peroxidase (GPX), which catalyzes the reduction of many oxidants, including H_2_O_2_ and peroxynitrite (ONOO^−^) (Panday et al., 2020), was found in all the strains tested except for *S. maltophilia* HZ34. Another is the timely repair of the cysteine and methionine residues that have been damaged by ROS oxidation. The disulfide isomerase (DsbC) and methionine sulfoxide reductase (Msr) systems, which can repair oxidized cysteine and methionine residues, respectively (Darby et al., 1998; Grimaud et al., 2001). The *dsbC*, *msrA* and *msrB* genes were found in all 213 strains.

Bacteria usually adopt two main strategies to maintain their osmotic balance (Gunde-Cimerman et al., 2018). One is to take up and accumulate a large amount of potassium (Vyrides and Stuckey, 2017). All 213 strains of *Stenotrophomonas* were found to have K^+^ uptake transporter proteins genes, including the Kdp-ATPase system (*kdp*), the potassium efflux system (*kef*), and the potassium transport system gene (*kup*) (Sleator and Hill, 2002). Another strategy relies on the biosynthesis and accumulation of compatible solutes (Figure 3), including sugars, such as trehalose; amino acids, such as proline and glutamate; and their derivatives, such as betaine (Zhao et al., 2023). Glycine betaine, a common compatible solute, can be synthesized by choline dehydrogenase (BetA) and betaine-aldehyde dehydrogenase (BetB) (Lamark et al., 1991). The *betA* gene was detected in 211 strains and was only absent in *S. rhizophila* GN_RF3 and *S. maltophilia* HZ34. In contrast, the *betB* gene was identified in 208 strains and only absent in five strains of *S. acidaminiphila*. Trehalose can be produced from UDP-glucose via the OstA-OstB pathway (Paul et al., 2008). The *ostA* and *ostB* genes were only absent in *S. maltophilia* HZ34. In addition, the glutamate (*gdhA*, *gltBD* and *glnA*) and proline (*proABC*) biosynthetic genes were detected in the genomes of all 213 strains of *Stenotrophomonas*. These results indicate that all the strains of *Stenotrophomonas* can eliminate ROS and tolerate osmotic stress, which endows them with the ability to survive more effectively in extreme environments.

### The antibiotic resistome of the strains of *Stenotrophomonas*

Antibiotics are used extensively to treat bacterial infections in agriculture and animal husbandry. Bacteria usually harbor many antibiotic resistance genes (ARGs) to survive (Zhuang et al., 2021). The number of ARGs per strain of *Stenotrophomonas* ranged from 3 to 23. Among them, *Stenotrophomonas* sp. strain SH_67_7 harbored the most ARGs, and *S. nitrodeiducens* CN18_13_7_16_R1_B_68_91 harbored the least (Supplementary Table S5). There were somewhat more ARGs in the clinical strains (average 14 per strain) than in the environmental strains (average 11 per strain), and *S. maltophilia* (8–23), *S. geniculata* (9–20), and *S. pavanii* (15–16) species had dramatically higher number of ARGs than the other species (4–13). The antibiotic efflux pumps genes, including the resistance-nodulation-cell division (RND) type and the major facilitator superfamily (MFS) type, were identified in all the strains of *Stenotrophomonas*. *rsmA,* an RND efflux pump gene involved in the resistance to fluoroquinolones, diaminopyrimidines and phenol antibiotics, was found in all 213 strains except for *S. geniculata* NWUBe21 and *S. rhizophila* DE0483. *smeDEF,* an RND efflux pump gene cluster involved in the resistance to macrolide, fluoroquinolone, tetracycline, and phenol antibiotics (Alonso and Martínez, 2000), was identified in 201 strains of *Stenotrophomonas*. Another RND efflux pump gene cluster *smeABC*, which is involved in resistance to aminoglycoside, β-lactam, and fluoroquinolone antibiotics (Li et al., 2002), was found in most of the clinical strains (124) and a minority of the environmental strains (28).

The resistance of bacteria to aminoglycosides (AGs) is primarily caused by AG-modifying enzymes (AMEs), which include *O*-phosphotransferases (APHs), *N*-acetyltransferases (AACs) and *O*-adenyltransferases (ANTs) (Wright, 1999). The *aph* gene was identified in 134 clinical strains and 53 environmental strains, while the *aac* gene was found in 59 clinical and 17 environmental strains. In particular, the *aph(9)-Ic*, *aph(3′)-IIc*, *aph(3″)-Ib*, *aph(6)-Id*, *aph(3′)-IIa*, *aph(3′)-*VIa, and *aph(6)-Ic* genes that encode variants of APH were identified in 185, 183, 5, 4, 1, 1 and 1 strains, respectively, while the *aac(6′)-Iz*, *aac(6′)-Ib8*, *aac(6′)-Iak*, *aac(6′)-Iap* and *aac(6′)-31* genes that encode variants of AAC were identified in 62, 12, 8, 3 and 1 strains, respectively. The *ant(2″)-Ia* gene was only found in *S. maltophilia* CV_2005, *S. maltophilia* 476BLSM_MAD, *S. maltophilia* 212 and *S. maltophilia* Smal28. In the *Stenotrophomonas* resistome, the highest numbers of types of genes for resistance were associated with aminocoumarin, phenicol, and tetracycline antibiotics, with 25, 14, and 12, respectively, while sulfonamide, monobactam, and mupirocin-like had the fewest types of resistance genes. The results indicated that the strains of *Stenotrophomonas* have multidrug resistance that can be adapted to respond to antibiotic pressure in the external environment.

### Tolerance to heavy metals

Most heavy metals are lethal in the environment (Gadd, 2010). The CopAB proteins are responsible for resistance to copper. In particular, CopA can bind copper ions in the periplasm to sequester the copper, while CopB is a transmembrane protein that can serve as an exporter of copper (Ge et al., 2021). The *copA* gene was identified in all 213 strains of *Stenotrophomonas*, and the *copB* gene was found in 212 strains (Figure 4). The CzcABC-type efflux pump proteins are involved in exporting cobalt, zinc and cadmium from the cells (Hussain et al., 2022). All 213 strains of *Stenotrophomonas* also harbored *czcA*, *czcB* and *czcC* genes. The *ars* operon ensures that various bacteria are resistant to arsenic. Some bacteria can reduce As(V) (arsenate) to As(III) (arsenite), which is catalyzed by arsenate reductase (ArsC). The As(III) that is produced can be directly pumped out of the cell via ArsB. In addition, MAs(III) (methylarsenite) can be oxidized to the less toxic MAs(V) (methylarsenate) via ArsH (Hao et al., 2021). The *arsC, arsB* and *arsH* genes were identified in 213, 209 and 194 genomes of *Stenotrophomonas*, respectively. The *chrR* gene encodes chromate reductase, which catalyzes the conversion of soluble and toxic Cr(VI) to insoluble and less toxic Cr(III), respectively (Baldiris et al., 2018), was found in all strains. In contrast, the *chrA* gene that encodes the chromate efflux transport protein, which pumps chromate out of the cytoplasm (Díaz-Pérez et al., 2007), was found in 55 strains. These genes in strains of *Stenotrophomonas* endow the bacteria with tolerance to heavy metals in the environment.

### Mobile genetic elements in *Stenotrophomonas*

MGEs are the direct evidence of the evolution and adaptation of microbial populations through the horizontal transfer of genes (Sobecky and Hazen, 2009). The MGEs in microbes usually include prophages, GIs, and insertion sequence (IS). A total of 637 prophage regions, including 79 questionable, 354 incomplete, and 204 intact prophages, were detected in these *Stenotrophomonas* genomes, thus, indicating that inducible or transferable functional prophages are widely distributed in strains of *Stenotrophomonas*. The number of prophages in each strain ranged from one to nine. Among them, 204 intact prophages were found in 60.6% (129/213 strains) of the strains of *Stenotrophomonas*, and they ranged from one to five intact prophages per strain (Supplementary Table S6). In particular, the largest proportion was composed of *Verrucomicrobia* phage P862 (NC_029047) followed by *Burkholderia* phage phi1026b (NC_005284), *Vibrio* phages VHML (NC_004456) and vB_VpaM_MAR (NC_019722). The major proteins of the prophages that were detected were the structural proteins of tail, capsid, portal, and head, and the functional enzymes terminase, integrase and lysin.

A total of 6,663 GIs were predicted in the 213 *Stenotrophomonas* strains. The number of GIs per strain ranged from 17 to 60 (Supplementary Table S1). *S. rhizophila* JC1 contained the highest number of GIs, while *S. chelatiphaga* DSM 21508^T^ contained the fewest (17). There are many vital functional protein genes in these GIs, including kinases (201 strains), MFS transporters (192 strains), GNAT family *N*-acetyltransferase (171 strains), ATP-binding proteins (166 strains), response regulator transcription factor (156 strains), efflux RND transporter (118 strains), siderophore receptor (112 strains), RNA polymerase (111 strains), nucleotidyl transferase (101 strains), DNA polymerase III subunit beta (70 strains), DNA replication/repair protein RecF (64 strains), lysozyme (54 strains), type II toxin-antitoxin system (52 strains), CusA/CzcA family heavy metal efflux RND transporter (68 strains), copper resistance protein (62 strains), arsenical resistance protein (40 strains), mercury reductase (31 strains) and chromate efflux transporter (25 strains). These genes are involved in the physiology at the transcriptional and translational levels, as well as confer pathogenicity, drug and heavy metal resistance to the strain (Supplementary Table S7). In addition, some Gls include the genes that encode integrases (205 strains), transposases (185 strains) and recombinases (163 strains), which may mediate the movement of GIs among the host bacteria. Thus, the prophages and GIs enhanced the genetic diversity of the *Stenotrophomonas* strains and enabled them to rapidly adapt to diverse ecological niches.

### Colonization potential and biofilm formation of *Stenotrophomonas* strains

Colonization of the rhizosphere by bacterial strains is the first and the foremost step. Genes involved in motility, chemotaxis, adhesion and biofilm formation are thought to contribute to this colonization (Kandel et al., 2017). The two site-specific tyrosine recombinases XerC and XerD are associated with competitive colonization on the root surface (Martínez-Granero et al., 2005). The two genes *xerC* and *xerD* were both found in the genomes of all 213 strains of *Stenotrophomonas* (Figure 4). The SmeDEF efflux pump, which is responsible for the microbial resistance to quinolones, has been found to be involved in the endophytic colonization of plant roots (García-León et al., 2014). The *smeDEF* gene was identified in all 213 strains. In addition, the *thuA* gene, which is involved in the utilization of trehalose, enhances the ability of the strain to colonize plants at the early stages (Pinski et al., 2020). The *thuA* gene was detected in 15 strains, including 10 *S. rhizophila* strains, *S. bentonitica* DSM 103927^T^, *S. bentonitica* VV6, *S. chelatiphaga* DSM 21508^T^, *S. tumulicola* JCM 30961^T^ and *Stenotrophomonas* sp. AP15.006.

As a physical barrier, biofilm can protect the embedded bacteria. Thus, the ability of bacteria to form biofilms determines their ability to colonize the root surface. Cellulose is an important component of biofilms in some species. The *bcsA* and *wssD* genes are two genes that are responsible for the biosynthesis of cellulose. Their inactivation significantly reduced the formation of biofilm and decreased the ability of strains to colonize the plant host (Barak et al., 2007; Monteiro et al., 2012). However, these two genes were only found in 43 strains of *Stenotrophomonas* (21 environmental and 22 clinical strains). In addition, lipopolysaccharides (LPS) and exopolysaccharides (EPS) facilitate the attachment of bacterial cells to the root surface (Kandel et al., 2017). The *lapA* gene, a key gene responsible for the assembly of LPS, was found in 210 strains. Rhamnose is an important subcomponent of LPS. The *rfbABCD* gene, a cluster that is responsible for the biosynthesis of rhamnose (Balsanelli et al., 2010), was found in all the strains of *Stenotrophomonas*. The *exoD* gene that encodes the protein for the biosynthesis of EPS (Reed and Walker, 1991) was found in 212 strains. The *gumD* gene, which is also responsible for the catalysis of the biosynthesis of EPS, (Meneses et al., 2011) was found in the genomes of 50 strains of *Stenotrophomonas* (33 environmental and 17 clinical strains). The results of genomic analyses showed that all the strains of *Stenotrophomonas* have a range of biofilm formation genes that can promote their ability to colonize plants. In addition, the ability to form biofilms has also been shown to be one of the main pathogenesis-related virulence factors (Wall et al., 2019). Inhibiting the expression of virulence-associated genes is reportedly correlated with the attenuation of the biofilm formation ability of *S. maltophilia* (Kim et al., 2018).

The formation of biofilm and cell adhesion are usually regulated by QS at the bacterial community level. The diffusible signal factor (DSF) is a QS molecule that can regulate the genes related to plant colonization, such those involved in chemotaxis, motility, the formation of biofilm and production of the multidrug efflux pump (Alavi et al., 2013a). The *rpf* gene cluster, which encodes all the components of the DSF system, was detected in 213 strains of *Stenotrophomonas* (Figure 4). Carbamoyl phosphate synthase, which is encoded by the *carAB* genes, can be used to degrade DSF (Newman et al., 2008). The *carA* gene was found in 212 strains (*S. maltophilia* HZ34 lacked), while the *carB* gene was found in 211. *S. maltophilia* HZ34 and *Stenotrophomonas* sp. SH_67_7 lack this gene. *pcoI* is a key gene related to the population sensing signals. Its deletion led to significant deficiencies in the formation of biofilm at the wheat rhizosphere (Wei and Zhang, 2006). The *pcoI* gene was only found in seven environmental strains, including five strains of *S. acidaminiphila*, and *S. nitritireducens* SCN18_13_7_16_R1_B_68_91 and *Stenotrophomonas* sp. MES1.

Some prevalent bacterial transcriptional regulators are also associated with plant-rhizobacterial interactions. The sigma-28 factor encoded by the *filA* gene is involved in the regulation of the expression of flagellin, chemotaxis, and motility-related genes (Yi et al., 2017). The *filA* gene was identified in the genomes of all the strains of *Stenotrophomonas*. The *greA* gene, another important transcription factor, determines the ability of host to adapt to hyperosmolarity and salt stress. The strains that lack this gene cannot establish an effective symbiotic relationship with plants (Nogales et al., 2002). The *greA* gene was found in 212 strains and was only absent from *S. maltophilia* HZ34. Cyclic diguanylate (c-di-GMP), a universal secondary messenger in bacteria, is involved in the regulation of a variety of physiological functions, including cell differentiation, biofilm formation, motility, adhesion, colonization of host tissues and the generation of pathogenic factors (Pinski et al., 2020). The biosynthesis of c-di-GMP is catalyzed by the gene that encodes diguanylate cyclase, which was found in 205 strains of *Stenotrophomonas*. The results indicate that strains of *Stenotrophomonas* have a range of transcriptional regulators that are related to the colonization of plants.

### Genes for the biosynthesis of polyamines and phytohormones and their diversity in strains of *Stenotrophomonas*

Polyamines, including putrescine, spermidine, spermine and cadaverine, may be involved in the promotion and protection of plant growth (Couée et al., 2004; Xie et al., 2014). Bacteria could synthesize putrescine from arginine step by step catalyzing by arginine decarboxylase (SpeA), agmatine deiminase (AguA), and N-carbamoyl-putrescine amidase (AguB) (Burrell et al., 2010; Michael, 2016) (Figure 3). The *speA*, *aguA*, and *aguB* genes were present in 213, 212, and 212 strains, respectively. Only *S. maltophilia* HZ34 lacked both the *aguA* and *aguB* genes (Figure 4). The *speE* gene, which is responsible for the conversion of putrescine to spermidine (Lee et al., 2012), was found in all the strains of *Stenotrophomonas*. The MetK and SpeD enzymes are responsible for the conversion of methionine to S-adenosyl-methioninamine (dcSAM), which is required for the biosynthesis of spermine (Xie et al., 2014). The *metK* and *speD* genes are found in all 213 strains. In addition, lysine decarboxylase (LdcC) catalyzes the conversion of L-lysine to cadaverine (Liu et al., 2022). The *ldcC* gene was found in the genomes of 203 strains of *Stenotrophomonas*. The presence of these genes suggests that strains of *Stenotrophomonas* can produce multiple types of polyamines.

IAA and cytokinin (CK) are the two most common phytohormones that influence various traits of plant growth and development, including cell division and elongation, seed germination, fruit development and the delay of senescence (Grossmann, 2009; McSteen, 2010; Akhtar et al., 2020). There are five different pathways for the biosynthesis of IAA from tryptophan. Among them, the indole-3-acetamide pathway (IAM), the indole-3-pyruvic acid pathway (IPA/IPyA) and the tryptamine pathway (TAM) are the most common pathways in bacteria (Zhang et al., 2019; Tang et al., 2023). The vital genes for the biosynthesis of tryptophan, including *trpAB, trpC, trpD, trpEG,* and *trpF*, were present in all 213 genomes of *Stenotrophomonas*. For the IPA pathway, the gene that is responsible for the conversion of tryptophan to IAAld was not detected in all 213 genomes, but the gene for indole-3-acetaldehyde dehydrogenase (AldA) that converts IAAld to IAA was found in all these genomes (Figure 3). In addition, the *iroA* gene that encodes indole-pyruvate ferredoxin oxidoreductase (IorA), which can directly convert IPA to IAA (Imada et al., 2017), was identified in 207 strains. As the IAM pathway, all 213 strains lacked the tryptophan monooxygenase that catalyzes the initial conversion of tryptophan to IAM, but the amidase gene responsible for the conversion of IAM to IAA was detected in all 213 genomes. For the TAM and TSO (tryptophan side-chain oxidase) pathway, only the *aldA* gene of the final step was detected, but the gene responsible for the conversion of tryptophan to IAAld was not detected.

The *miaA* gene encodes a tRNA dimethylallyltransferase that is responsible for the conversion of dimethylallyl diphosphate to N_6_-(dimethylallyl) adenosine (iPR). The *miaB* gene, which encodes tRNA-2-methylthio-N(6)-dimethylallyladenosine synthase, converts iPR to 2-methylthio-N_6_-(dimethylallyl)adenosine (2MeSiPR). Both genes are involved in the biosynthesis and conversion of CK (Nascimento et al., 2020) and were identified in all the *Stenotrophomonas* genomes except for *S. rhizophila* IS26 and *Stenotrophomonas* SH_67_7, which both lacked *miaB*.

### Phosphate-solubilization and the acquisition of iron

Phosphorus (P) is an essential macronutrient for plant growth and development. Although there are adequate amounts of P in the soil, most of the organic and inorganic phosphate that is found in the soil is not directly available to plants. Many bacteria that are associated with plants can solubilize P into a form that can be utilized by plants (Sharma et al., 2013). Organic phosphates are primarily degraded by the enzyme acid phosphatases, alkaline phosphatases and phytase that are produced by bacteria (Sharma et al., 2013), while inorganic phosphates can be solubilized by the production of organic acids, such as gluconic acid, citric acid, malic acid, and succinic acid (Vassilev et al., 2006). There are two categories of phosphatases that are based on their optimal pH, including acid phosphatase and alkaline phosphatase, which both dephosphorylate phosphate ester or the phosphoric anhydride bonds in organic compounds (Jorquera et al., 2008). The acid phosphatase and alkaline phosphatase genes were found in 213 and 197 strains, respectively (Figure 4). The phytase encoded by *phyA* is responsible for the catalysis of the degradation of phytate to release P, and this gene was identified in 212 strains. *S. nitritireducens* SCN18_13_7_16_R1_B_68_91 does not produce *phyA*. In addition, the *gdh* gene encodes a phosphate starvation-inducible glucose dehydrogenase (GDH), which may be involved in the solubilization of mineral phosphate (Sharma et al., 2005). This gene was identified in 205 strains of *Stenotrophomonas*. Among them, five *S. acidaminiphila* strains, *S. pictorum* JCM 9942^T^, *Stenotrophomonas* sp. MES1 and *S. maltophilia* HZ34 lacked *gdh* gene. An exopolyphosphatase and an inorganic pyrophosphatase are encoded by the *ppx* and *ppa* genes, respectively. They catalyze the hydrolysis of inorganic pyrophosphate. Both genes were found in all 213 genomes of *Stenotrophomonas*. These genes enable these strains of *Stenotrophomonas* to solubilize soil phosphate, which provides adequate amounts of P for their own metabolism and that of their host plant.

Similar to P, iron is an essential nutrient for plant and microbial growth, but its bioavailability is limited owing to the low solubility of ferric oxide (Fe^3+^) ions (Behnke and LaRoche, 2020). Siderophores, iron chelators that are produced by microorganisms, can convert the iron into soluble complexes that can be utilized by plants (Ahmed and Holmström, 2014). The comparative analysis revealed that most strains of *Stenotrophomonas* possessed the genes to biosynthesize catecholate siderophores. In particular, 26 and 189 strains possessed the catecholate siderophore biosynthetic genes *fiu* and *cirA*, respectively, and 171 and 187 strains had the biosynthetic genes *entAFH* and *fepA*, respectively, for the catecholate siderophore enterobactin (Patzer et al., 2003; Pakarian and Pawelek, 2016). In Gram-negative bacteria, the uptake and transfer of iron ions (Fe^3+^) by siderophores is powered by the Ton complex (TonB-ExbB-ExbD) (Faraldo-Gómez and Sansom, 2003). The *tonB*, *exbB* and *exbD* genes were present in all 213 genomes (Figure 4). In addition, many bacteria can take up ferrous iron (Fe^2+^) using the Feo system under anaerobic and acidic environments (Lau et al., 2016). The Feo system is primarily composed of FeoA and FeoB, (Kalidasan et al., 2018). The *feoB* gene was detected in all 213 strains of *Stenotrophomonas* except for *S. maltophilia* HZ34, while the *feoA* gene was not found in *Stenotrophomonas* SH_67_7 and *S. maltophilia* HZ34. The results suggest that the strains of *Stenotrophomonas* can enhance the availability of Fe^3+^ in an environment deficient in iron, which benefits both their survival and the growth of plant.

### Potential of the genus *Stenotrophomonas* for biocontrol

The biological control of microbes in the rhizosphere provides their hosts with natural protection from soilborne phytopathogens (Pandit et al., 2022). The production of cell wall-degrading enzymes by rhizobacteria is one of the primary mechanisms that they use to destroy pathogens. The chitinase encoded by the *chiA* gene can degrade the chitin in the cell walls of pathogenic fungi (Kharade and McBride, 2014). The *chiA* gene was detected in 204 genomes of *Stenotrophomonas*. In addition, the chitin-binding proteins (CBPs), encoded by the *cbpD* gene, can increase the efficiency of some specific chitinases (Manjeet et al., 2013). The *cbpD* gene is found in 183 strains of *Stenotrophomonas* (Figure 4). Proteases and lipases also play an important role in biocontrol (Friedrich et al., 2012). Among them, protease genes were identified in 205 strains of *Stenotrophomonas*, while lipase genes were identified in 200 strains. Six strains lacked the chitinase, protease, and lipase genes, including *S. acidaminiphila* T0-18, *S. acidaminiphila* T25-65 and *S. nitritireducens* SCN18_13_7_16_R1_B_68_91 isolated from bioreactors, *S. pictorum* JCM 9942^T^ isolated from the soil and *S. acidaminiphila* SPDF1 isolated from cell culture media.

Polyhydroxybutyrate (PHB), the most abundant and well-characterized polymer of bacterial polyhydroxyalkanoate (PHA) (Jendrossek, 2009), has proven to have both antibacterial and antifungal activities (Ma et al., 2019). The biosynthesis of PHB is a three-step process that begins with acetyl-CoA acetyltransferase (PhaA), which catalyzes the condensation of two molecules of acetyl-CoA to acetoacetyl-CoA; this compound is then reduced to 3-hydroxybutyryl-CoA by acetoacetyl-CoA reductase (PhaB) and finally catalyzed by PhaC (poly(3-hydroxyalkanoate) polymerase) to produce PHB (Jendrossek and Pfeiffer, 2014). The three genes are found in the genomes of all strains of *Stenotrophomonas*. The keratinases KerSMD and KerSMF have broad substrate specificity and can be used as pesticides (Gupta and Ramnani, 2006; Yue et al., 2011). Both genes *kerSMD* and *kerSMF* were detected in 205 strains of *Stenotrophomonas*. The results suggest that *Stenotrophomonas* may be a useful tool for the integrated management of various plant pathogens and pests.

## Discussion

**Figure 5:**
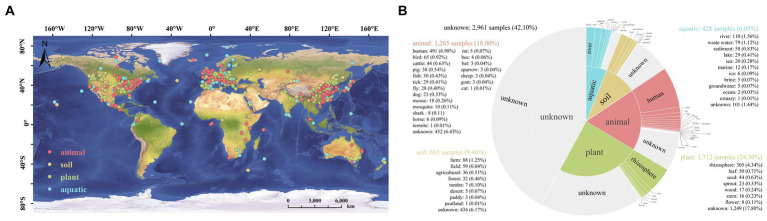
Biogeographic distribution analysis of the genus *Stenotrophomonas* based on the Microbe Atlas Project (MAP) database and pipeline. **(A)** The global distribution of *Stenotrophomonas*. **(B)** Number of samples that contain the representative OTU sequence per habitat and sub-habitat. OTU, operational taxonomic unit.

The genus *Stenotrophomonas* is widely distributed in various habitants on the globe, including plant, animal, soil and aquatic environments (Figure 5A). Among them, strains of *Stenotrophomonas* were detected in 1,712 plant samples (24.3%), while only 428 samples were detected in aquatic environments (6.09%); the rhizosphere is the primary habitat among plant environments that has been shown to harbor these bacteria (Figure 5B). This suggests that the strains of the genus are closely associated with plants. Previous studies have also shown that strains of *Stenotrophomonas* can promote plant growth, as well as manage pests and pathogens (Kumar et al., 2023). However, there have been no systematic analyses of the abilities of this genus to adapt to the environment and promote the growth of plants. To our knowledge, this is the first study that systematically and comprehensively elucidates the possible adaptive and plant-promoting mechanisms of the genus *Stenotrophomonas* using the comparative genomic method.

*Stenotrophomonas* was named for its tolerance to oligotrophic conditions. The genomic analysis showed that almost all of the strains that have been analyzed can synthesize all 20 amino acids, which suggests that most of them can grow well in simple nutrients. In addition, the comparative results suggest that the strains of this genus are extremely effective at utilizing carbon sources. In detail, nearly all of the *Stenotrophomonas* strains that have been analyzed harbor the GH3-family cellulose-degrading genes and the GH2- and GH31-family genes for hemicellulose hydrolase. Most of the strains also contained intact EMP and TCA pathways to utilize the monomer carbohydrates. This indicates that the strains of *Stenotrophomonas* can not only degrade two types of abundant carbohydrates in the biosphere, including both cellulose and hemicellulose, but also their monomers. The high ability of these strains to utilize carbon sources enables these them to easily survive in the soil. Alternatively, all the strains of *Stenotrophomonas* contain SOD and CAT genes to scavenge excess ROS, as well as the *dsbC* and *msrAB* genes to repair the cysteine and methionine residues that been damaged owing to oxidation by ROS. These genes ensure that the strains of *Stenotrophomonas* can remove the ROS produced during their rapid growth on eutrophic conditions, which suggests that the strains of this genus can also thrive in eutrophic media.

Extreme conditions, including osmotic pressure, antibiotics and heavy metals, are another important limiting factor for the survival of the strain in the environment. All the strains of *Stenotrophomonas* possess the mechanism to pump in potassium ions using a specific transport system (*kdp, kup* and *kef*) to maintain their osmotic balance. In addition, most of them also harbored genes to synthesize compatible solutes, such as betaine, trehalose, glutamate and proline, to manage osmotic stress. The numerous genes for antibiotic resistance that are present in the genomes increase the ability of *Stenotrophomonas* to survive in high-stress environments that contain antibiotics. Previous studies have shown that SmeABC and SmeDEF are the two most common efflux pumps in *S. maltophilia* (Wang et al., 2018). The results of this study confirmed these two efflux pumps are common not only in *S. maltophilia* but also in the entire genus of *Stenotrophomonas*. In addition, all the genomes of *Stenotrophomonas* harbored AG-modifying enzyme genes, including APHs and AACs, which enable their resistance to broad-spectrum aminoglycoside antibiotics. It is notable that fewer strains of *Stenotrophomonas* harbor the genes for resistance to monobactam, mupirocin and sulfonamide antibiotics, which suggests that they could be used to select clinical candidates and develop them to some extent. *cop*, *czc, ars* and *chr* operon units were identified in all the genomes of *Stenotrophomonas*, and they provide resistance to copper, cobalt, zinc, cadmium, arsenic and chromium. In summary, the genes related to carbon source utilization, antioxidative stress, osmotic protection, antibiotic and heavy metal resistance enhance the ability of the strains of *Stenotrophomonas* to survive in extreme environments, as well as to provide a foundation for its colonization of the rhizosphere and promotion of plant growth.

Chemotaxis and motility are vitally important for free-living bacteria because the levels of nutrients vary, and nutrients are often present as point sources over time (Blackburn et al., 1998). All the strains of *Stenotrophomonas* have chemotaxis and motile device flagella, which suggests that these strains can sense a variety of physicochemical cues and rapidly move to inhabit a niche that is more conducive to their survival. Furthermore, the active motility facilitated by the flagella and guided by chemotactic responses promotes the initial contact of strains of *Stenotrophomonas* with their host root surface, which increases the efficiency of colonization. Similarly, *Stenotrophomonas* can form biofilms that increase their adhesion to biotic and abiotic surfaces and result in their resistance to heat, antibiotics, UV and other environmental stresses, which leads to a promotion in the colonization and biocontrol efficacy of roots (Shaheen et al., 2010; Gao et al., 2019). A series of transcriptional regulators were identified in all the genomes of *Stenotrophomonas* that regulate and enhance the levels of expression of the genes related to motility, chemotaxis, adhesion, and biofilm formation to improve the survival of these strains and their efficiency of colonization. Altogether, the presence of genes related to motility, chemotaxis and biofilm formation contribute to the resistance of strains of *Stenotrophomonas* to environmental stresses and facilitate their efficient colonization of plant hosts.

**Figure 6:**
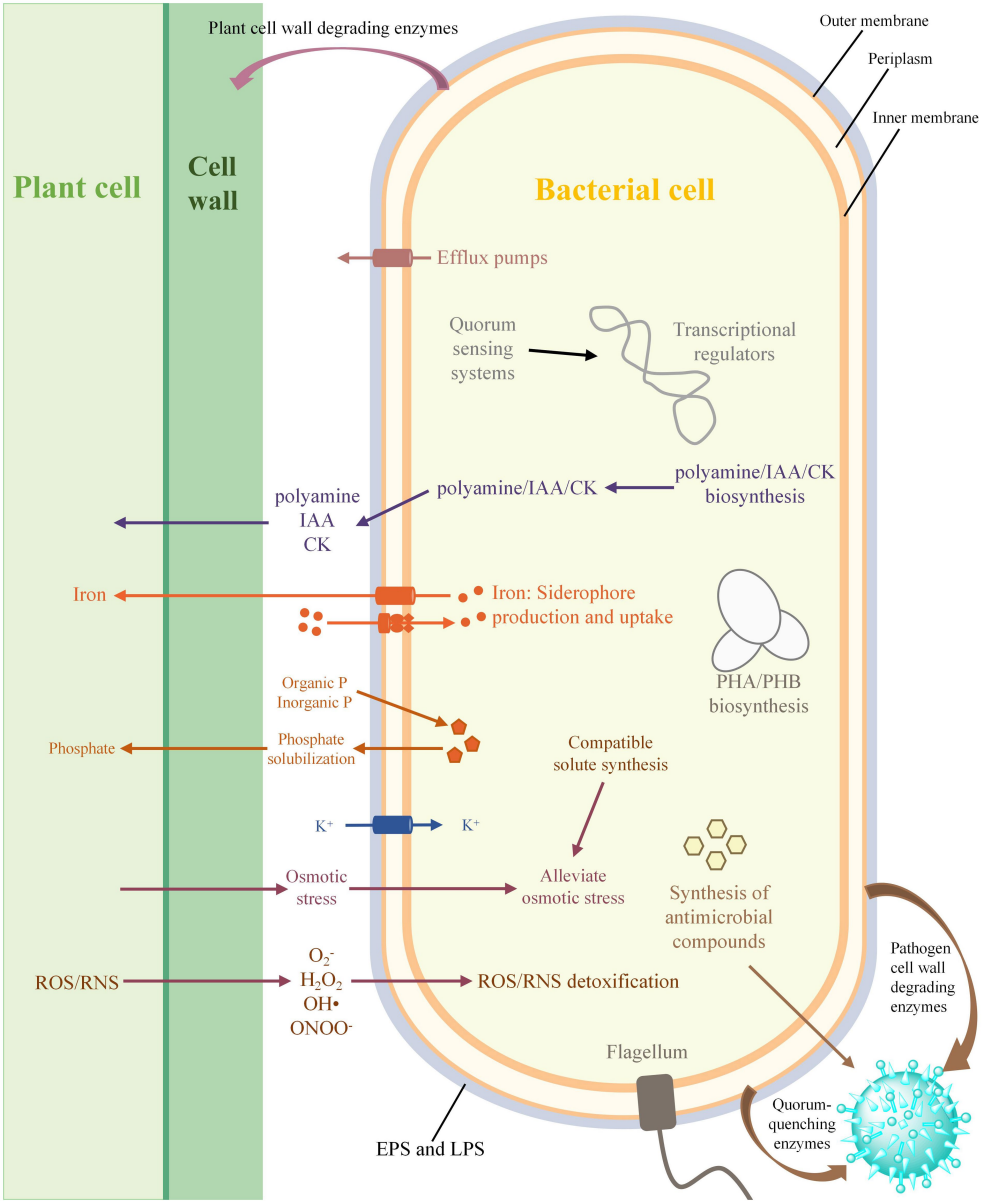
Schematic diagram of plant-growth promoting bacterial interactions. IAA, indole-3-acetic acid; CK, cytokinin; EPS, exopolysaccharide; LPS, lipopolysaccharide; PHA, polyhydroxyalkanoate; PHB, polyhydroxybutyrate; ROS, reactive oxygen species; RNS, reactive nitrogen species.

Rhizobacteria can promote the growth of host plants through a variety of direct and indirect mechanisms, with direct mechanisms that include the biosynthesis of phytohormones and the facilitation of acquiring resources (Figure 6). The genomic analysis revealed that all the strains of *Stenotrophomonas* harbor the genes involved in the biosynthesis of L-tryptophan and the final step of IAA biosynthesis. This result was supported by a previous study that showed that *Stenotrophomonas* sp. 169 and *S. maltophilia* R551-3 can produce IAA, but the genes that catalyze the first two steps of IAA biosynthesis were not found in their genomes (Taghavi et al., 2009; Ulrich et al., 2021). This suggests that *Stenotrophomonas* may have unidentified enzymes involved in the initial conversion of tryptophan or the existence of novel pathways for the biosynthesis of IAA. The *miaAB* gene for the biosynthesis of cytokinin and several genes involved in the biosynthesis of polyamines, such as putrescine, spermidine and cadaverine, were also identified in all the genomes of *Stenotrophomonas*. Polyamines are not only associated with the establishment of biological interactions between the roots and rhizosphere microorganisms (Jang et al., 2002; Alavi et al., 2013a,b), but they also protect plants from various environmental stresses, including acidic, oxidative, cold and osmotic stresses (Kasukabe et al., 2004; Alavi et al., 2013a). In addition, each of the 213 *Stenotrophomonas* genomes analyzed harbored multiple genes that were responsible for phosphate-solubilization, which suggests that the genus has a strong ability to solubilize phosphate to meet the plant requirements for P. All the strains of *Stenotrophomonas* can also secrete a catecholate siderophore to chelate Fe^3+^ and utilize the Feo system to absorb Fe^2+^. These iron acquisition systems also play an important role in biological control in addition to providing iron to plants because they can reduce availability of iron to plant pathogens and limit their growth in the rhizosphere by promoting the uptake of iron (Aznar et al., 2015).

*Stenotrophomonas* can also indirectly promote the growth of plants by inhibiting phytopathogens, which is primarily accomplished through the production of antimicrobial compounds and cell wall-degrading enzymes and quenching the QS of pathogens (Figure 6). In particular, strains of *Stenotrophomonas* harbor many abundant BGCs with PiPP-like, arylpolyene, and NRP-metallophore/NRPS. The secondary metabolites produced by these BGCs may contribute to the prevention of plant damage by phytopathogens and enhance the competition for niche resources (Bulgarelli et al., 2013). All the strains of *Stenotrophomonas* can also synthesize PHB, which not only has antifungal and antibacterial properties, but also serves as carbon and energy storage compounds in bacteria that can improve their survival and tolerance to stress during starvation (Sadykov et al., 2017). In addition, the genes that encode various hydrolytic enzymes, such as keratinases, chitinases, proteases and lipases, were identified in most of the *Stenotrophomonas* genome. Previous studies have shown that keratinases can be used as effective biocontrol agents for plant-parasitic nematodes (Pinski et al., 2020), while chitinases, proteases, and lipases can protect plants against phytopathogens by destroying the cell walls of fungi (Zhang et al., 2001). The *carAB* gene required for the rapid degradation of DSF was also found in most strains of *Stenotrophomonas*, which controls these pathogens that produce DSF by quenching their QS abilities (Newman et al., 2008). Altogether, these findings suggest that *Stenotrophomonas* can have beneficial effects on plants through a variety of mechanisms.

**Figure 7:**
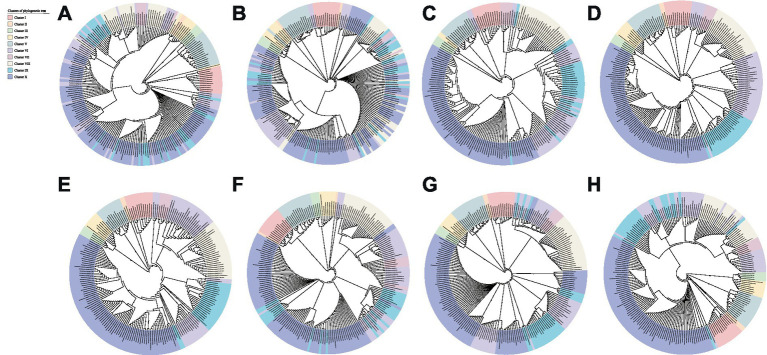
Phylogenetic tree of *Stenotrophomonas* constructed based on the amino acid sequences of AguB **(A)**, SpeE **(B)**, LdcC **(C)**, AmiE **(D)**, IroA **(E)**, AldA **(F)**, MiaA **(G)**, MiaB **(H)**.

MGEs may expand the species gene pool and enhance bacterial adaptation and competitiveness in specific habitats through a large number of mechanisms of horizontal gene transfer (HGT) (Dobrindt et al., 2004). Different numbers of horizontally transferred GIs and prophages were found in each *Stenotrophomonas* genome, and genes involved in the translocation and catabolism of various substrates, siderophore receptors, and resistance to antibiotics and heavy metals were identified in the GIs, which indicates that many strains of *Stenotrophomonas* have acquired the genes for resistance to antibiotics and heavy metals through HGT. In addition, the phylogenetic analysis revealed that the biosynthetic genes for polyamines (*aguB*, *speE*, and *ldcC*), IAA (*iroA* and *aldA*) and cytokinins (*miaA* and *miaB*) could have been acquired by HGT. In particular, the topology of the phylogenetic trees based on the sequences of AguB (Figure 7A), SpeE (Figure 7B), LdcC (Figure 7C), IroA (Figure 7E), AldA (Figure 7F), MiaA (Figure 7G), and MiaB (Figure 7H) differed significantly from that based on the core genome (Figure 1A). Strains that clustered together in the core genome tree were usually scattered in different branches in the trees that were constructed using single AguB, SpeE, LdcC, IroA, AldA, MiaA, and MiaB proteins. In contrast, the topology of the tree based on AmiE (Figure 7D) is similar to that of the core genome-based phylogenetic tree with only minor differences. The *S. maltophilia* strains RC09, NEC1, KMM_349, E824, NS26, and 291 located in Cluster VI in the core phylogenetic tree scattered separately in different clusters on the AimE tree. *S. maltophilia* PC315, which is a member of Cluster VI on the core genome tree, presents as Cluster IX in the AmiE tree. Although the phylogenetic tree based on the AmiE sequence differed slightly from the core genome phylogenetic tree in topology, the G + C content of the *amiE* gene (68.1–74.9%) is significantly higher than that of the host genome (63.8–69.3%) (Supplementary Figure S3D). This suggests that the *amiE* gene may also have been acquired through HGT. In addition, the open pan-genome and increasing number of novel genes suggest that the strains of *Stenotrophomonas* can introduce alien genes by the exchange of genetic material with other community members in their habitat and promote their broader genomic plasticity.

In summary, the genus *Stenotrophomonas* has a high degree of environmental adaptability and may promote plant growth through one or more mechanisms, which indicate that this genus is a promising PGPB, and can be used as a viable alternative to synthetic pesticides and fertilizers in agriculture to some extent. Thus, this bacterium could provide an effective and environmentally friendly solution to address agricultural pollution and ensure food security. Moreover, the presence of large numbers of heavy metal resistance genes in *Stenotrophomonas* proves that it may have immense potential in assisting in the phytoremediation of heavy metal pollution. However, it is worth noting that the diversity of *Stenotrophomonas* strains makes it difficult to distinguish characteristics because of their advantageous interactions with plants and their facultatively harmful infections with humans (Berg and Martinez, 2015). Therefore, advanced molecular techniques should be developed to identify and discriminate between human pathogenic and non-pathogenic strains of *Stenotrophomonas*, so that non-pathogenic strains can be applied in future agricultural systems.

## Conclusion

The comparative genomic analysis of 213 strains of *Stenotrophomonas* revealed that despite their geographical isolation and diverse hosts, genetic factors related to environmental adaptation and plant promotion were identified in all of them. In particular, nearly all the strains can utilize ubiquitous carbohydrates in the biosphere, such as cellulose and hemicellulose, as carbon and energy sources. The presence of functional genes associated with antioxidant stress, osmotic pressure protection, heavy metal and antibiotic resistance, motility, chemotaxis and biofilm formation enable them to colonize and survive in a variety of host environments. The genes involved in dissolving phosphates and producing siderophores, polyamines, phytohormones, antimicrobial compounds, and pathogen cell wall-degrading enzymes confer the *Stenotrophomonas* strain with plant growth-promoting and biocontrol properties. Overall, the results of this study suggest that *Stenotrophomonas* may have immense potential for use in sustainable agricultural practices and bioremediation technologies and can be used as a suitable candidate for a microbial biocontrol agent or an anti-stress agent for crops.

## Data availability statement

The original contributions presented in the study are included in the article/Supplementary material, further inquiries can be directed to the corresponding author.

## Author contributions

YZ: Conceptualization, Data curation, Investigation, Writing – original draft, Writing – review & editing, Visualization. W-JD: Investigation, Writing – review & editing. LX: Investigation, Writing – review & editing. J-QS: Conceptualization, Funding acquisition, Writing – review & editing.

## Footnotes

^1^ https://microbeatlas.org/

^2^ https://itol.embl.de/

^3^ http://ggdc.dsmz.de/ggdc.php

^4^ https://antismash.secondarymetabolites.org/

^5^ https://bcb.unl.edu/dbCAN2

^6^ http://blast.ncbi.nlm.nih.gov/Blast.cgi

^7^ https://card.mcmaster.ca/

^8^ http://phaster.ca/

^9^ http://www.pathogenomics.sfu.ca/islandviewer/

## Glossary

ANI: average nucleotide identity
dDDH: digital DNA–DNA hybridization
COG: clusters of Orthologous Groups of proteins
smBGCs: secondary metabolite biosynthesis gene clusters
SodB: superoxide dismutase [Fe]
SodC: superoxide dismutase [Cu-Zn]
KatE: catalase
AhpC: alkylhydroperoxidase
Bcp: thioredoxin peroxidase
DsbC: disulfide interchange protein
MsrA: peptide methionine-S-sulfoxide reductase
MsrB: peptide methionine-R-sulfoxide reductase
OtsA: trehalose-6-phosphate synthase
OtsB: trehalose 6-phosphate phosphatase
ProA: glutamate-5-semialdehyde dehydrogenase
ProB: glutamate 5-kinase
ProC: pyrroline-5-Carboxylic acid reductase
GdhA: glutamate dehydrogenase
GlnA: glutamine synthetase
GltB: glutamate synthase large chain
GltD: glutamate synthase small chain
CopA: copper-exporting P-type ATPase A
CopB: copper-transporting P-type ATPase B
CzcABC: cobalt-zinc-cadmium resistance protein
ArsB: arsenical pump membrane protein
ArsH: arsenical resistance protein
LapA: Lipopolysaccharide assembly protein A
RfbA: glucose-1-phosphate thymidylyltransferase
RfbB: dTDP-glucose 4,6-dehydratase
RfbC: dTDP-4-dehydrorhamnose 3,5-epimerase
RfbD: dTDP-4-dehydrorhamnose reductase
GumD: exopolysaccharide xanthan biosynthesis glycosyltransferase
RpfC: DSF synthase
RpfC: sensor histidine kinase
RpfG: two-component system response regulator
PcoI: acyl-homoserine-lactone synthase
SpeE: spermidine synthase
MetK: methionine adenosyltransferase
SpeD: S-adenosylmethionine decarboxylase proenzyme
Fiu and CirA: catecholate siderophore receptor
EntA: enterotoxin type A
EntF: enterobactin synthase component F
EntH: enterobactin synthase component H
FepA: ferrienterobactin receptor
TonB: TonB-dependent siderophore receptor
ExbB: TonB-system energizer
ExbD: biopolymer transporter
Feo: Fe^2+^ transport protein
