# Supplementary figures and images for: A comprehensive comparative genomic analysis revealed that plant growth promoting traits are ubiquitous in strains of Stenotrophomonas
Source: Front Microbiol. 2024 May 16;15:1395477. doi: 10.3389/fmicb.2024.1395477 (PMC11138164; doi:10.3389/fmicb.2024.1395477)

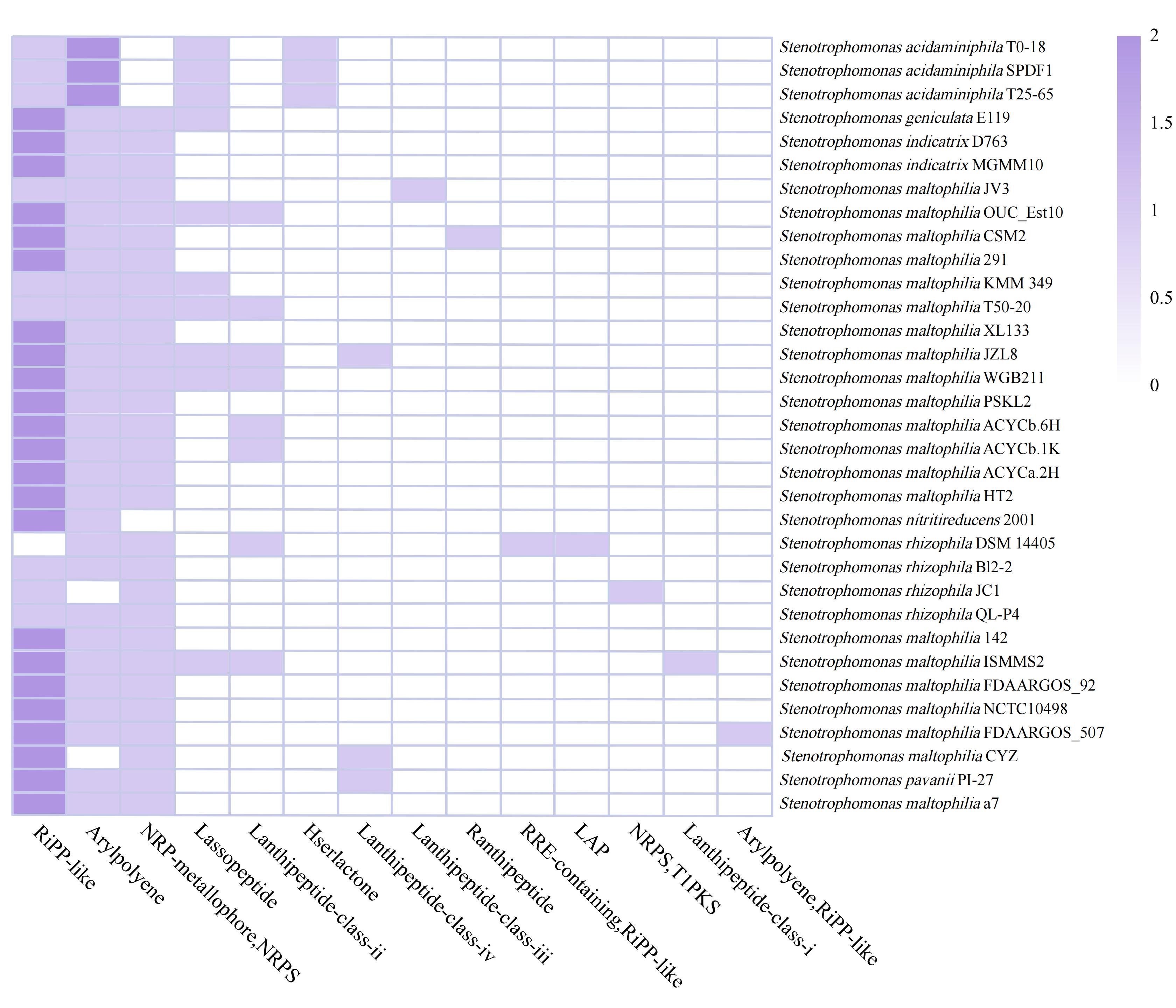

Supplement: Supplementary file 1 [file Image_1.JPEG]

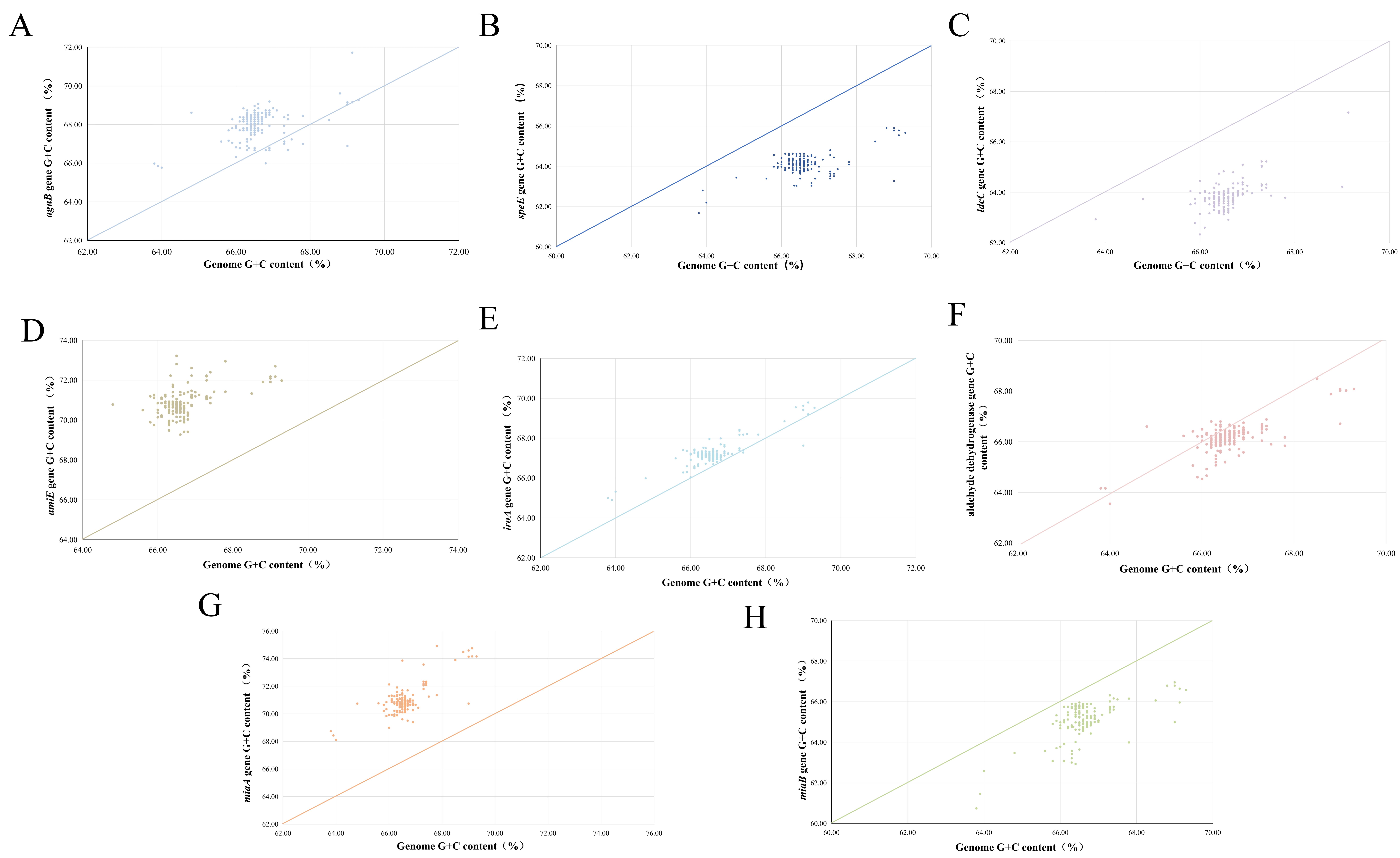

Supplement: Supplementary file 3 [file Image_3.JPEG]
